# The Relationship Between the Elastic Properties and Pain Pressure Threshold in Cesarean Scar Tissue—An Observational Study

**DOI:** 10.3390/healthcare12212166

**Published:** 2024-10-31

**Authors:** Ana González-Muñoz, Leo Pruimboom, Santiago Navarro-Ledesma

**Affiliations:** 1 Clinical Medicine and Public Health PhD Program, Faculty of Health Sciences, University of Granada, Av. de la Ilustración, 60, 18071 Granada, Spain; 2 Clinica Actium, Avenida Hernan Nuñez de Toledo 6, 29018 Malaga, Spain; 3 Department of Physiotherapy, University Chair in Clinical Psychoneuroimmunology (University of Granada and PNI Europe), 2518 JP The Hague, The Netherlands; leopruimboom@cpnieurope.com (L.P.); snl@ugr.es (S.N.-L.); 4 Department of Physiotherapy, Faculty of Health Sciences, Campus of Melilla, University of Granada, Querol Street 5, 52004 Melilla, Spain

**Keywords:** cesarean section, elastography, algometry, scar tissue, pain pressure thresholds

## Abstract

Background/Objectives: Cesarean section (C-section) scars can lead to chronic pain due to changes in tissue properties. Combining elastography and algometry can assess these scars comprehensively by identifying areas of increased rigidity and quantifying pain sensitivity. This study aims to evaluate the efficacy of using elastography and algometry together to assess cesarean scar tissue, correlating tissue stiffness with pain thresholds for targeted pain management. Methods: Thirty-one non-pregnant women who had undergone a C-section between six months and two years prior participated. Elastography measured tissue stiffness, while algometry provided quantitative measures of pain sensitivity on and around the scar. The correlation between tissue stiffness and pain thresholds was analyzed. Results: Elastography identified areas of increased rigidity within the scar tissue, which corresponded with lower pain thresholds measured by algometry, indicating higher pain sensitivity. A significant correlation was found between increased tissue stiffness and reduced pain thresholds (*p* < 0.05). Conclusions: Combining elastography and algometry offers a powerful diagnostic tool for evaluating C-section scars. This approach identifies areas that may benefit from targeted pain management interventions, enhancing understanding and treatment of pain associated with cesarean scars. Incorporating these techniques into clinical practice could improve patient outcomes and quality of life.

## 1. Introduction

Cesarean section (C-section) is a widely performed surgical procedure globally. Recent data from the World Health Organization (WHO) indicate that approximately 21% of global births were conducted via C-section in 2021, with significant regional variations, reaching over 40% in parts of Latin America and the Caribbean [1,2,3]. Cesarean birth rates continue to rise worldwide, with 2016 data reporting rates of 24.5% in Western Europe, 32% in North America, and 41% in South America [4]. In Spain, C-section rates exceed 24.5% in certain regions, especially within private healthcare settings, where patient preferences and financial incentives contribute to the higher rates compared to public hospitals [4,5]. While C-sections are crucial in preventing severe complications during childbirth, such as maternal or fetal death, the resulting scar tissue can lead to various symptoms and dysfunctions, including chronic pain, adhesions, and difficulties in subsequent surgeries [6,7].

Adhesions are one of the most common complications associated with C-section scars. The incidence of adhesions after a single C-section can reach 24%, rising to 59% in women who have undergone three or more C-sections [7,8]. Other risk factors for developing adhesions include maternal age over 35 years, obesity (BMI ≥ 30), infections, and the period during which the C-section was performed. Notably, surgeries performed between 1997 and 2013 showed higher adhesion rates compared to previous decades. This increase is likely due to changes in surgical practices and materials, such as the adoption of single-layer uterine closure and the use of chromic catgut sutures, which are associated with heightened inflammatory responses. Single-layer closure, while efficient, has been linked to increased inflammation and adhesion formation due to the increased exposure of the decidua, which can impair healing [7,9]. Chromic catgut, widely used during this period, also incites a stronger inflammatory response compared to synthetic alternatives like polyglactin 910 (Vicryl), further increasing adhesion risk [8,9]. Wound healing is further influenced by factors such as diabetes and the quality of connective tissue [7]. These adhesions can contribute to functional impairments, such as subfertility, chronic pelvic pain, and even complications during future surgical procedures [9].

Chronic pain after C-section is also a significant concern, with the prevalence ranging from 6% to 18%, depending on factors such as study design, follow-up periods, and definitions of chronic pain [10]. In a European cohort, including Spain, approximately 12% of women reported chronic pain six months post-C-section [11]. This pain may stem from a combination of vascular, inflammatory, and tensional changes in the scar tissue, which can affect sympathetic tone, blood flow, microcirculation, and mechanotransduction, all contributing to chronic pain [12].

One of the key issues in addressing post-C-section pain is the quality of the scar tissue itself. Di Pascuo et al. observed that C-section scar stiffness is higher than that of the surrounding myometrium, which can be attributed to histological changes such as reduced smooth muscle cell density and increased type III collagen content [13]. This increased stiffness can restrict tissue mobility, potentially contributing to the formation of myofascial adhesions, which are known to cause pain and limit functional movement [14].

In clinical practice, evaluating the quality of scar tissue is crucial, as poor-quality scar tissue can lead to various dysfunctions and negatively impact the patient’s quality of life. For pain assessment, the pressure pain threshold (PPT) is commonly used to evaluate sensitivity at characteristic painful points associated with C-section scars [12]. On the other hand, the stiffness of the scar tissue can be assessed through elastography, a non-invasive ultrasound technique that measures tissue elasticity by evaluating its stiffness and deformation under pressure [15]. Elastography provides valuable information about the mechanical properties of the scar, which could be directly related to the patient’s perception of pain and overall tissue health [12].

There is growing interest in studying the relationship between the mechanical properties of scar tissue, as measured by elastography, and the patient’s perception of pain, assessed through the PPT. Understanding this relationship could have important clinical implications, as it may provide insights into how tissue quality influences pain perception and functional outcomes. A stiffer scar, which may indicate poorer tissue quality, could be associated with greater pain and dysfunction, potentially leading to a reduced quality of life. Therefore, investigating tools that objectively assess both pain perception and tissue stiffness is vital for improving clinical outcomes in patients with C-section scars [12,15,16].

In physiotherapy, various techniques are employed to improve scar tissue elasticity, including manual therapy, radiofrequency, and ultrasound-guided invasive neuromodulation [17]. These interventions aim to restore tissue mobility and reduce myofascial adhesions, which, if untreated, can persist and significantly impact motor function. A precise evaluation of the scar is essential for the effective application of these therapeutic techniques. Elastography is particularly useful in this context, as it provides an objective measure of tissue stiffness, which can guide treatment decisions and monitor progress [12,15,18].

This study aims to analyze the association between elastography measurements in C-section scars and the pressure pain threshold at specific painful points observed in patients with C-section scars. By exploring this relationship, we seek to enhance our understanding of how tissue stiffness and pain perception are linked, which could have significant implications for clinical practice and patient care.

## 2. Material and Methods

### 2.1. Design

This study was a cross-sectional observational study with ethical approval from the Human Research Ethics Committee of the University of Málaga, Spain (approval number CEUMA: 42-2024-H), reported according to the STROBE declaration and conducted in accordance with the Declaration of Helsinki.

### 2.2. Setting

Patients were recruited at a physiotherapy private clinic in Malaga from September 2023 to December 2023. Information about the study was provided, including information sheets and informed consent from all subjects.

### 2.3. Participants

A total of 31 patients participated in the study.

The inclusion criteria were (i) participants aged 30–46 years; (ii) patients who underwent one or more transverse C-section surgeries, with a scar older than six months and younger than 2 years; (iii) patients with a post-C-section scar with a fibrotic appearance and possible surrounding fascial restrictions; and (iv) patients with a pain score above 5 on the visual analog scale (VAS).

The exclusion criteria were (i) patients with neurological, inflammatory, or orthopedic injuries that impair balance, hearing, vision, or cognitive abilities necessary to answer questions or complete questionnaires; (ii) patients with other types of injuries in the area to be treated; (iii) patients with local problems that could reduce skin elasticity (e.g., hyperkeratosis); and (iv) patients with keloids on the scar.

### 2.4. Recruitment Procedures

During their first visit to the physiotherapy clinic, all participants were informed and asked to sign the informed consent. After obtaining the information and agreeing to participate, all subjects underwent the evaluations.

### 2.5. Outcomes Measures

#### 2.5.1. Primary Outcome: Pain Pressure Threshold

Algometry is an objective measurement procedure that quantifies the pressure pain threshold (PPT) to which a patient responds. This procedure is performed using an algometer, a device specifically designed to measure the pressure applied to a selected anatomical area accurately. Thus, a correlation is established between pain perception and the functionality of a particular structure [19]. Algometric measurements were performed using the Pain Test FPX algometer (Wagner Instruments, Riverside, CT, USA). Pain thresholds were defined in kg/cm^2^, with pressure applied at a constant rate. All measurements were recorded by a specialized physiotherapy researcher trained to apply a constant speed of 50 kPa/s, with approximately five years of experience in algometry measurements. Pain thresholds were measured in myofascial tension areas of the C-section scar. The scar was divided into three parts, and in each third, the point of greatest myofascial mobility restriction was identified for algometry measurement.

Each measurement was conducted with the subject in a supine position, applying the device at a 90-degree angle to the skin surface. The subject was asked to indicate “STOP” when they felt the first clear sensation of pain at the test point. A trial measurement was conducted on the forearm muscles to familiarize the subject with the pain threshold sensation before measuring the actual points. The same investigator recorded the result after each measurement. Measurements were repeated three times.

#### 2.5.2. Secondary Outcome: Elastography

The Logiq S7 with a 6–15 MHz linear probe (GE Healthcare, Milwaukee, WI, USA) was used for all measurements by an expert physiotherapist with eleven years of experience in ultrasound imaging. Tissue quality evaluation was performed by pixel quantification using software included in the ultrasound equipment.

In this study, strain elastography (SEL) was used, which produces axial deformation parallel to the externally applied force on the body surface using the ultrasound probe. This results in a quantitative evaluation of stiffness [20]. All participants were positioned in the same orientation used for the protocol. The patient lay supine on the table with legs extended along the body. The therapist was positioned to the patient’s right, using their right hand to maneuver the ultrasound probe. Pain thresholds were measured in myofascial tension areas of the C-section scar. The scar was divided into three parts, and in each third, the point of greatest myofascial mobility restriction was identified for elastographic evaluation.

### 2.6. Statistical Analysis

Statistical analysis was performed using SPSS Statistics Version 24 for Windows (IBM Corporation, Armonk, NY, USA). Pearson correlation coefficients were calculated to evaluate the linear relationship between elastography and PPT measurements. This analysis was performed separately for each location. Furthermore, simple linear regression models were constructed to quantify the relationship between elastography (independent variable) and the PPT (dependent variable) for each body location. The models were evaluated based on their R-squared values and the statistical significance of the coefficients.

### 2.7. Sample Size

The G*Power 3.1.9.7 software was used to calculate the required sample size based on a significance level (α) of 0.05 and a statistical power (1-β) of 0.80, anticipating a moderate effect size (r = 0.3). This calculation initially indicated a required sample size of 85 participants to detect significant correlations between tissue elasticity and the pain pressure threshold (PPT) in cesarean scars. However, due to practical recruitment limitations, the final sample consisted of 31 participants, which remains a valid cohort for exploratory analysis. Comparable research has demonstrated the clinical relevance of moderate sample sizes in studies exploring tissue stiffness and pain sensitivity. For instance, Seven et al. identified a significant relationship between increased cesarean scar stiffness and pain sensitivity using a sample of 34, suggesting that moderate sample sizes can effectively detect meaningful clinical correlations in scar tissue research. Similarly, Navarro-Ledesma et al. reported correlations between stiffness and PPT in fibromyalgia patients with a sample size of 30, underscoring the applicability of elastography and PPT assessments in smaller cohorts [6,12]. The final sample of 31 participants thus allows for a preliminary analysis of the association between scar elasticity and the PPT, aligning with the standards established by related studies.

## 3. Results

**Figure 1:**
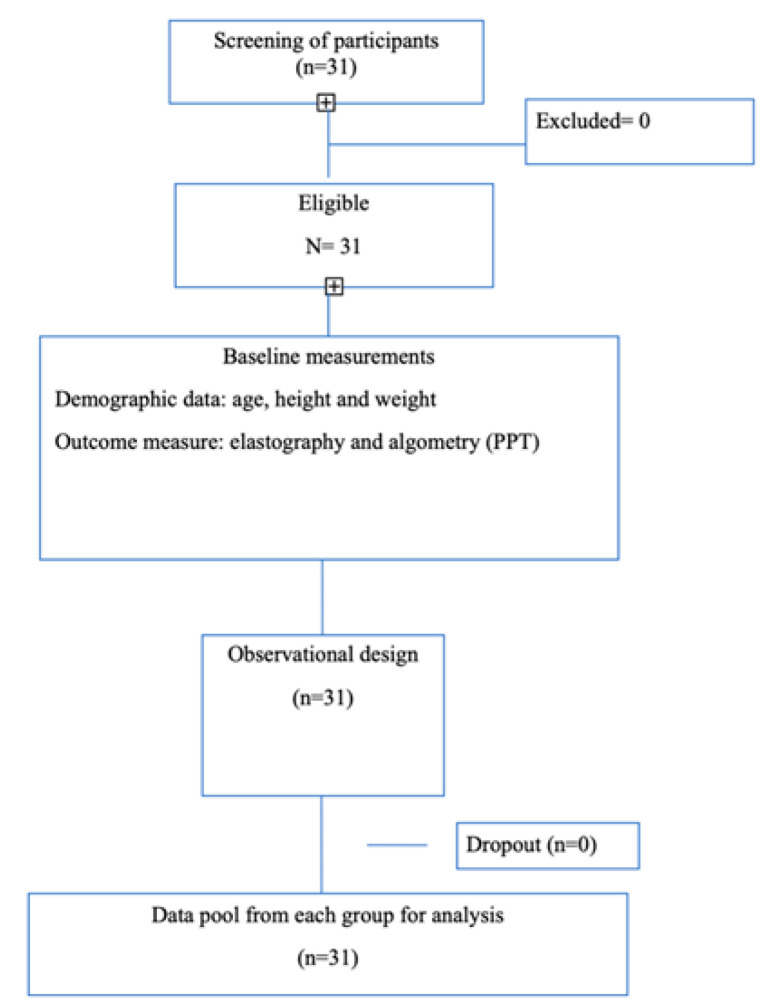
Flow diagram of participants.

A total of 31 non-pregnant women who had undergone a transverse C-section between six months and two years prior participated in the study. The demographic characteristics of the participants are detailed in Table 1 and Table 2, showing variables such as height, age, weight, algometry and elastography measurements (see Figure 1).

### 3.1. Relationship Between the Elastic Properties of Tissue and Pain Pressure Thresholds in the Cesarean Scar Tissue

A small but significant level of correlation (*p* = 0.03; r = 0.386) between the elastic properties of tissue and the pain pressure thresholds at the first point of measurement in the targeted cesarean scar tissue was found. The rest of the points assessed along the cesarean scar tissue showed no correlation (*p* = 0.103; r = 0.588, and *p* = 0.410; r = 0.159) (see Table 3).

### 3.2. Left-Side Regression Analysis

**Figure 2:**
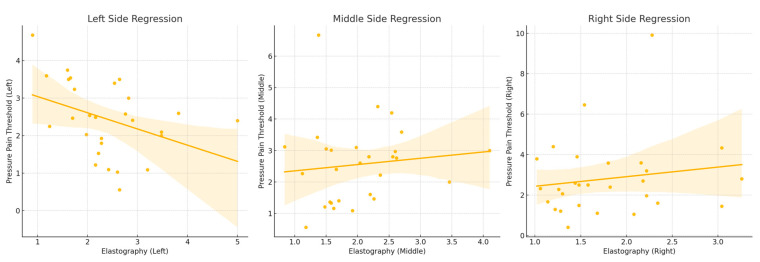
Note: The regression analysis results indicate a significant negative relationship between elastography measurements and PPT values on the left side, suggesting that increased tissue stiffness is associated with a decreased pain threshold. However, no significant relationships were identified for the middle and right sides, indicating that the observed associations might be location-specific.

The regression analysis for the left side revealed a statistically significant model with an R-squared value of 0.148. This indicates that approximately 14.8% of the variability in PPT values can be explained by elastography measurements. The model yielded a significant F-statistic of 4.534 (*p* = 0.0429), suggesting that the regression model provides a better fit than a model with no predictors. The regression coefficient for elastography was -0.4321, with a *p*-value of 0.043. This negative coefficient indicates that higher elastography values are associated with lower PPT values on the left side, implying that increased tissue stiffness is linked to a decreased pain threshold (see Figure 2).

### 3.3. Middle-Part Regression Analysis

For the middle side, the regression model exhibited an R-squared value of 0.024, signifying that only 2.4% of the variability in PPT values could be explained by elastography measurements. The F-statistic for this model was 0.6335, with a corresponding *p*-value of 0.4295, indicating that the model was not statistically significant. The regression coefficient for elastography was 0.4741, with a *p*-value of 0.433. This positive coefficient suggests a potential positive relationship between elastography values and PPT, but the lack of statistical significance undermines the reliability of this observation (see Figure 2).

### 3.4. Right-Side Regression Analysis

The regression analysis for the right side yielded an R-squared value of 0.024, indicating that elastography measurements explained only 2.4% of the variability in PPT values. The model’s F-statistic was 0.6335, with a *p*-value of 0.433, demonstrating that the model was not statistically significant. The regression coefficient for elastography was 0.4741, with a *p*-value of 0.433. Similarly to the middle side, this positive coefficient indicates a potential positive relationship between elastography values and PPT; however, the lack of statistical significance limits the confidence in this finding (see Figure 2).

## 4. Discussion

The primary goal of this study was to explore the association between elastic changes in C-section scars, measured via elastography, and pressure pain thresholds (PPTs). Our results showed a statistically significant but moderate correlation between PPT and scar elasticity (SEL) on the left side of the scar (*p* = 0.03; r = 0.386). However, no significant correlations were observed for the middle or right side of the scar. This finding highlights the local variability in scar tissue characteristics, potentially influenced by factors such as the surgeon’s positioning during the procedure and the suturing technique used.

### 4.1. Comparison with the Existing Literature

To our knowledge, this is the first study to assess the relationship between elastography and PPT specifically in C-section scars, making comparisons with the existing literature somewhat challenging. Nonetheless, our results align with previous research indicating that increased tissue stiffness correlates with higher pain sensitivity in other contexts. For instance, Navarro-Ledesma et al. (2023) found similar associations between tissue elasticity and pain pressure thresholds in fibromyalgia patients, further supporting the notion that stiffer tissues tend to have lower pain thresholds [12]. Similarly, Seven et al. (2020) demonstrated that increased stiffness in subcutaneous C-section scar tissue can predict intra-abdominal adhesions, which adds to the validity of using elastography in scar assessments [6].

Additionally, scar stiffness increases during pregnancy, especially in the second trimester. Combining quantitative elastography with algometry has revealed that areas of greater stiffness tend to have lower pain thresholds, emphasizing the importance of evaluating both stiffness and pain sensitivity for more effective post-surgical pain management. This methodology is not only effective for identifying problematic areas but also for guiding personalized therapeutic interventions, improving the clinical management of patients with C-section scars [10,21].

However, unlike prior studies that generally reported uniform tissue stiffness across the scar, our analysis uncovered regional differences within the scar. Specifically, no significant correlations were observed in the middle and right parts of the scar (*p* = 0.103; r = 0.588 and *p* = 0.410; r = 0.159, respectively). This localized variation suggests that scar stiffness, and consequently pain, may vary within different regions of the same scar, possibly due to the surgeon’s technique or dominant hand positioning during the operation. This is consistent with research by Roberge et al. (2011) and Ahn et al. (2016), who highlighted the impact of the suturing technique and suture material on scar formation and tissue stiffness [22,23].

### 4.2. Clinical Utility and Implications

The findings from this study have important clinical implications, particularly in the management and treatment of pain in C-section scars. The significant relationship between higher SEL values (indicating greater tissue stiffness) and lower PPTs on the left side of the scar suggests that elastography can be a useful tool for identifying areas of increased stiffness that may be associated with heightened pain sensitivity. This association between tissue stiffness and pain has been observed in various other clinical contexts, such as in musculoskeletal disorders [12,15,24]. In clinical practice, integrating SEL measurements into routine scar assessments could help clinicians better understand the biomechanical properties of scar tissue, allowing for more targeted interventions, such as manual therapy or other techniques aimed at improving tissue elasticity and reducing pain.

The relationship between SEL and PPTs observed in this study suggests that stiffer areas of the scar may be more prone to painful sensitivity, potentially due to alterations in tissue tensegrity and mechanotransduction. When tissue stiffness is high, it may affect how mechanical stimuli are transmitted through the tissue, leading to altered pain responses. This insight supports the use of quantitative elastography as an objective measure to identify and monitor areas of the scar that may require more focused pain management strategies. Additionally, combining elastography with PPT assessments could enhance the precision of post-surgical treatment plans, ensuring that therapeutic interventions are both individualized and effective for improving patient outcomes.

### 4.3. Surgical Techniques and Material

Suturing techniques play a crucial role in scar tissue development and associated pain outcomes. Various suture techniques are used in C-sections, each with advantages and disadvantages in terms of healing and adhesion formation. Single-layer suturing involves one suture line, closing the uterus in one step, which can be continuous or interrupted. Single-layer suturing, particularly locked sutures, has been linked to a higher risk of adhesion formation and uterine rupture due to the inclusion of decidua, which negatively impacts healing [22,25,26]. In contrast, double-layer suturing involves a first layer closing the myometrium without including the decidua, followed by a second layer reinforcing the initial closure. This technique results in better healing and fewer adhesions due to more uniform healing and the exclusion of the decidua from the suture line [23,27]. Additionally, suture material influences adhesion formation, with chromic catgut causing more inflammatory reactions and adhesions compared to polyglactin 910 (Vicryl), which is associated with faster healing and fewer adhesions [28].

These findings suggest that it would be worthwhile to further investigate how different suturing techniques and materials, as well as the surgeon’s dominant hand, the side on which the surgeon is positioned, and the side of initiation of the suture, contribute to variations in scar tension and postoperative pain, potentially causing greater mechanical tension due to traction on the surrounding soft tissues. This gap in the literature provides an opportunity for future research to improve patient outcomes.

Therefore, it would be very interesting to conduct studies evaluating the side on which surgeons start and finish suturing wounds and the dominant upper limb to analyze the prevalence of increased scar tension in correlation with this work’s methodology due to the lack of scientific literature correlating these data.

### 4.4. Limitations and Future Research

One of the primary limitations of our study was the sample size. Although our results are significant, the lower number of participants limits the generalizability of the findings. Recruiting a larger, more diverse sample would enhance the power of future studies. Additionally, the localized nature of the correlation observed suggests that other factors, such as surgical technique and patient-specific variables, may play a role in scar stiffness and pain perception. These factors should be explored in greater detail in future research, ideally through longitudinal studies that follow patients over time to track changes in tissue stiffness and pain thresholds.

Moreover, further validation of elastography as a reliable tool for assessing scar tissue in a clinical setting is needed. Although elastography has proven useful in other areas of medicine, including breast, thyroid, and prostate evaluations [12,29,30], its application in gynecology, specifically for C-section scars, remains limited. However, despite the benefits of this tool and its cost-effectiveness compared to other evaluation methods, there are still limitations in its applicability due to intra- and inter-observer variability, particularly for scars [31]. Expanding its use, alongside tools like algometry for pain assessment, could provide a more comprehensive approach to managing C-section scar-related pain.

## 5. Conclusions

In conclusion, our study provides preliminary evidence of a relationship between tissue stiffness, as measured by elastography, and pain perception in C-section scars. The significant correlation on the left side of the scar suggests that tissue elasticity may influence pain sensitivity, though the variability across different regions of the scar warrants further investigation. While our findings are promising, larger-scale studies with more comprehensive data are necessary to confirm these initial observations and better understand the mechanisms underlying scar-related pain. By continuing to explore these relationships, we can develop more targeted, effective interventions for managing post-C-section pain.

## Figures and Tables

**Table 1:** Demographics.

Variable	N	Missing	Mean	95% CI (Lower)	95% CI (Upper)	SD	Minimum	Maximum	Shapiro–Wilk W	Shapiro–Wilk *p*-Value
Height	31	0	1.65	1.63	1.67	0.0512	1.50	1.75	0.968	0.458
Age	30	1	35.9	34.5	37.4	3.81	29	46	0.964	0.388
Weight	31	0	65.7	61.6	69.9	11.2	48.0	89.0	0.942	0.091

The CI of the mean assumes that the sample means follow a t-distribution with N − 1 degrees of freedom. CI = Confidence Interval.

**Table 2:** Algometry and elastography measurements.

	N	Missing	Mean	95% CI (Lower)	95% CI (Upper)	SD	Minimum	Maximum	Shapiro–Wilk W	Shapiro–Wilk *p*-Value
Algometry left	31	0	2.38	2.02	2.73	0.966	0.560	4.68	0.975	0.670
Algometry middle	31	0	2.47	2.02	2.92	1.23	0.560	6.67	0.906	0.010
Algometry right	31	0	2.74	2.08	3.41	1.82	0.410	9.91	0.791	<0.001
Elasto left	30	1	2.40	2.08	2.72	0.847	0.900	5.00	0.951	0.176
Elasto middle	30	1	2.02	1.76	2.28	0.701	0.840	4.10	0.934	0.062
Elasto right	29	2	1.78	1.55	2.01	0.607	1.02	3.26	0.895	0.008

The CI of the mean assumes that the sample means follow a t-distribution with N − 1 degrees of freedom. CI = Confidence Interval.

**Table 3:** Level of correlation between SEL and PPT.

	SCAR Left Side	SCAR Middle Side	SCAR Right Side
Relationship SEL-PPT	r = 0.386 *p* = 0.035	r = 0.103 *p*= 0.588	r = 0.159 *p* = 0.410

SEL = strain elastography; PPT = pain pressure threshold.

## Data Availability

All data associated with this study are present in the paper. All requests for other materials will be reviewed by the corresponding author to verify whether the request is subject to any intellectual property or confidentiality obligations.
